# Commonalities across computational workflows for uncovering explanatory variants in undiagnosed cases

**DOI:** 10.1038/s41436-020-01084-8

**Published:** 2021-02-12

**Authors:** Shilpa Nadimpalli Kobren, Dustin Baldridge, Matt Velinder, Joel B. Krier, Kimberly LeBlanc, Cecilia Esteves, Barbara N. Pusey, Stephan Züchner, Elizabeth Blue, Hane Lee, Alden Huang, Lisa Bastarache, Anna Bican, Joy Cogan, Shruti Marwaha, Anna Alkelai, David R. Murdock, Pengfei Liu, Daniel J. Wegner, Alexander J. Paul, Undiagnosed Diseases Network, Maria T. Acosta, Margaret Adam, David R. Adams, Pankaj B. Agrawal, Mercedes E. Alejandro, Justin Alvey, Laura Amendola, Ashley Andrews, Euan A. Ashley, Mahshid S. Azamian, Carlos A. Bacino, Guney Bademci, Eva Baker, Ashok Balasubramanyam, Dustin Baldridge, Jim Bale, Michael Bamshad, Deborah Barbouth, Pinar Bayrak-Toydemir, Anita Beck, Alan H. Beggs, Edward Behrens, Gill Bejerano, Jimmy Bennett, Beverly Berg-Rood, Jonathan A. Bernstein, Gerard T. Berry, Anna Bican, Stephanie Bivona, Elizabeth Blue, John Bohnsack, Carsten Bonnenmann, Devon Bonner, Lorenzo Botto, Brenna Boyd, Lauren C. Briere, Elly Brokamp, Gabrielle Brown, Elizabeth A. Burke, Lindsay C. Burrage, Manish J. Butte, Peter Byers, William E. Byrd, John Carey, Olveen Carrasquillo, Ta Chen Peter Chang, Sirisak Chanprasert, Hsiao-Tuan Chao, Gary D. Clark, Terra R. Coakley, Laurel A. Cobban, Joy D. Cogan, Matthew Coggins, F. Sessions Cole, Heather A. Colley, Cynthia M. Cooper, Heidi Cope, William J. Craigen, Andrew B. Crouse, Michael Cunningham, Precilla D’Souza, Hongzheng Dai, Surendra Dasari, Joie Davis, Jyoti G. Daya, Matthew Deardorff, Esteban C. Dell’Angelica, Shweta U. Dhar, Katrina Dipple, Daniel Doherty, Naghmeh Dorrani, Argenia L. Doss, Emilie D. Douine, David D. Draper, Laura Duncan, Dawn Earl, David J. Eckstein, Lisa T. Emrick, Christine M. Eng, Cecilia Esteves, Marni Falk, Liliana Fernandez, Carlos Ferreira, Elizabeth L. Fieg, Laurie C. Findley, Paul G. Fisher, Brent L. Fogel, Irman Forghani, Laure Fresard, William A. Gahl, Ian Glass, Bernadette Gochuico, Rena A. Godfrey, Katie Golden-Grant, Alica M. Goldman, Madison P. Goldrich, David B. Goldstein, Alana Grajewski, Catherine A. Groden, Irma Gutierrez, Sihoun Hahn, Rizwan Hamid, Neil A. Hanchard, Kelly Hassey, Nichole Hayes, Frances High, Anne Hing, Fuki M. Hisama, Ingrid A. Holm, Jason Hom, Martha Horike-Pyne, Alden Huang, Yong Huang, Laryssa Huryn, Rosario Isasi, Fariha Jamal, Gail P. Jarvik, Jeffrey Jarvik, Suman Jayadev, Lefkothea Karaviti, Jennifer Kennedy, Dana Kiley, Isaac S. Kohane, Jennefer N. Kohler, Susan Korrick, Mary Kozuira, Deborah Krakow, Donna M. Krasnewich, Elijah Kravets, Joel B. Krier, Grace L. LaMoure, Seema R. Lalani, Byron Lam, Christina Lam, Brendan C. Lanpher, Ian R. Lanza, Lea Latham, Kimberly LeBlanc, Brendan H. Lee, Hane Lee, Roy Levitt, Richard A. Lewis, Sharyn A. Lincoln, Pengfei Liu, Xue Zhong Liu, Nicola Longo, Sandra K. Loo, Joseph Loscalzo, Richard L. Maas, John MacDowall, Calum A. MacRae, Ellen F. Macnamara, Valerie V. Maduro, Marta M. Majcherska, Bryan C. Mak, May Christine V. Malicdan, Laura A. Mamounas, Teri A. Manolio, Rong Mao, Kenneth Maravilla, Thomas C. Markello, Ronit Marom, Gabor Marth, Beth A. Martin, Martin G. Martin, Julian A. Martinez-Agosto, Shruti Marwaha, Jacob McCauley, Allyn McConkie-Rosell, Colleen E. McCormack, Alexa T. McCray, Elisabeth McGee, Heather Mefford, J. Lawrence Merritt, Matthew Might, Ghayda Mirzaa, Eva Morava, Paolo M. Moretti, Paolo Moretti, Deborah Mosbrook-Davis, John J. Mulvihill, David R. Murdock, Anna Nagy, Mariko Nakano-Okuno, Avi Nath, Stanley F. Nelson, John H. Newman, Sarah K. Nicholas, Deborah Nickerson, Shirley Nieves-Rodriguez, Donna Novacic, Devin Oglesbee, James P. Orengo, Laura Pace, Stephen Pak, J. Carl Pallais, Christina G. S. Palmer, Jeanette C. Papp, Neil H. Parker, John A. Phillips, Jennifer E. Posey, Lorraine Potocki, Bradley Power, Barbara N. Pusey, Aaron Quinlan, Archana N. Raja, Deepak A. Rao, Wendy Raskind, Genecee Renteria, Chloe M. Reuter, Lynette Rives, Amy K. Robertson, Lance H. Rodan, Jill A. Rosenfeld, Natalie Rosenwasser, Francis Rossignol, Maura Ruzhnikov, Ralph Sacco, Jacinda B. Sampson, Susan L. Samson, Mario Saporta, Judy Schaechter, Timothy Schedl, Kelly Schoch, C. Ron Scott, Daryl A. Scott, Vandana Shashi, Jimann Shin, Rebecca H. Signer, Edwin K. Silverman, Janet S. Sinsheimer, Kathy Sisco, Edward C. Smith, Kevin S. Smith, Emily Solem, Lilianna Solnica-Krezel, Ben Solomon, Rebecca C. Spillmann, Joan M. Stoler, Jennifer A. Sullivan, Kathleen Sullivan, Angela Sun, Shirley Sutton, David A. Sweetser, Virginia Sybert, Holly K. Tabor, Amelia L. M. Tan, Queenie K.-G. Tan, Mustafa Tekin, Fred Telischi, Willa Thorson, Audrey Thurm, Cynthia J. Tifft, Camilo Toro, Alyssa A. Tran, Brianna M. Tucker, Tiina K. Urv, Adeline Vanderver, Matt Velinder, Dave Viskochil, Tiphanie P. Vogel, Colleen E. Wahl, Melissa Walker, Stephanie Wallace, Nicole M. Walley, Chris A. Walsh, Jennifer Wambach, Jijun Wan, Lee-kai Wang, Michael F. Wangler, Patricia A. Ward, Daniel Wegner, Mark Wener, Tara Wenger, Katherine Wesseling Perry, Monte Westerfield, Matthew T. Wheeler, Jordan Whitlock, Lynne A. Wolfe, Jeremy D. Woods, Shinya Yamamoto, John Yang, Muhammad Yousef, Diane B. Zastrow, Wadih Zein, Chunli Zhao, Stephan Zuchner, Shamil R. Sunyaev, Isaac S. Kohane

**Affiliations:** 1 Department of Biomedical Informatics, Harvard Medical School, Boston, MA USA; 2 Department of Pediatrics, Washington University School of Medicine, St. Louis, MO USA; 3 Center for Genomic Discovery, University of Utah, Salt Lake City, UT USA; 4 Division of Genetics, Brigham and Women’s Hospital, Harvard Medical School, Boston, MA USA; 5 National Human Genome Research Institute (NHGRI) at the National Institutes of Health (NIH), Bethesda, MD USA; 6 Department of Human Genetics and Hussman Institute for Human Genomics, University of Miami Health System, Miami, FL USA; 7 Division of Medical Genetics, Department of Medicine, University of Washington, Seattle, WA USA; 8 Department of Human Genetics, David Geffen School of Medicine at the University of California, Los Angeles, CA USA; 9 Department of Pathology and Laboratory Medicine, David Geffen School of Medicine at the University of California, Los Angeles, CA USA; 10 Department of Biomedical Informatics, Vanderbilt University Medical Center, Nashville, TN USA; 11 Stanford Center for Undiagnosed Diseases, Stanford, CA USA; 12 Institute for Genomic Medicine, Columbia University Medical Center, New York City, NY USA; 13 Department of Molecular and Human Genetics, Baylor College of Medicine, Houston, TX USA; 14 Baylor Genetics, Houston, TX USA; 15 McDonnell Genome Institute, Washington University School of Medicine, St. Louis, MO USA; 16 National Institutes of Health, Undiagnosed Diseases Program Clinical Site, Bethesda, MD USA; 17 University of Washington and Seattle Children’s Hospital Clinical Site, Seattle, WA USA; 18 Harvard-affiliated Boston Children’s Hospital, Massachusetts General Hospital, Brigham and Women’s Hospital, and Brigham Genomic Medicine Clinical Site, Boston, MA USA; 19 Baylor College of Medicine, Clinical Site, Houston, TX USA; 20 University of Utah Clinical Site, Salt Lake City, UT USA; 21 Stanford University Clinical Site, Stanford, CA USA; 22 University of Miami Clinical Site, Miami, FL USA; 23 Washington University of Saint Louis, Clinical Site, Saint Louis, MO USA; 24 Washington University of Saint Louis, Model Organism Screening Center, Saint Louis, MO USA; 25 Children’s Hospital of Philadelphia or University of Pennsylvania Clinical Site, Philadelphia, PA USA; 26 Vanderbilt University Clinical Site, Nashville, TN USA; 27 University of California, Los Angeles, Clinical Site, Los Angeles, CA USA; 28 University of Alabama Coordinating Center, Birmingham, AL USA; 29 Duke University Clinical Site, Durham, NC USA; 30 Mayo Clinic Metabolomics Core, Rochester, MN USA; 31 Baylor Genetics Sequencing Core, Houston, TX USA; 32 Harvard Medical School Coordinating Center, Boston, MA USA; 33 Columbia University Clinical Site, New York City, NY USA; 34 Baylor College of Medicine, Model Organism Screening Center, Houston, TX USA; 35 University of Oregon, Model Organism Screening Center, Eugene, OR USA

## Abstract

**Purpose:**

Genomic sequencing has become an increasingly powerful and relevant tool to be leveraged for the discovery of genetic aberrations underlying rare, Mendelian conditions. Although the computational tools incorporated into diagnostic workflows for this task are continually evolving and improving, we nevertheless sought to investigate commonalities across sequencing processing workflows to reveal consensus and standard practice tools and highlight exploratory analyses where technical and theoretical method improvements would be most impactful.

**Methods:**

We collected details regarding the computational approaches used by a genetic testing laboratory and 11 clinical research sites in the United States participating in the Undiagnosed Diseases Network via meetings with bioinformaticians, online survey forms, and analyses of internal protocols.

**Results:**

We found that tools for processing genomic sequencing data can be grouped into four distinct categories. Whereas well-established practices exist for initial variant calling and quality control steps, there is substantial divergence across sites in later stages for variant prioritization and multimodal data integration, demonstrating a diversity of approaches for solving the most mysterious undiagnosed cases.

**Conclusion:**

The largest differences across diagnostic workflows suggest that advances in structural variant detection, noncoding variant interpretation, and integration of additional biomedical data may be especially promising for solving chronically undiagnosed cases.

## INTRODUCTION

Next-generation exome sequencing (ES) and genome sequencing (GS) have revolutionized the process for diagnosing rare and novel genetic conditions.^1^ Traditionally, the diagnostic process has primarily been driven by phenotype, with clinicians comparing patients’ symptoms to others encountered in their prior experience and clinical training and/or to a knowledgebase of known human diseases.^2^ In a typical undiagnosed case, however, either a patient’s phenotype is not indicative of any known disease, or tests to confirm the presence of a suspected genetic condition are inconclusive. In these instances, ES and GS have enabled health-care providers to pursue a genetics-driven diagnostic approach in parallel, where the genetic variation uncovered in a patient can be assessed with respect to not only its known phenotypic associations^3^ but also to its prevalence in background populations,^4^ predicted pathogenicity,^5^ functional consequences, and mode of inheritance to reveal novel disease-causing loci. Indeed, while traditional clinical case review and directed diagnostic assays continue to solve difficult cases, ~74% of newly diagnosed genetic conditions have been attributed to analyses of ES and GS data.^6,7^ However, the diagnosis rate for patients with potentially unique genetic conditions is still ~35%,^7^ suggesting ample opportunity for methodological improvements to advance our understanding of the genetic underpinnings of phenotypic extremes.

With this goal in mind, cross-institutional initiatives such as Care4Rare in Canada (http://care4rare.ca) and Solve-RD in Europe (http://solve-rd.eu) have been established to connect and enable clinical researchers to uncover the genetic origins of disease in undiagnosed patients. In addition to furthering basic genetics research, these efforts have provided scores of patients with an end to diagnostic uncertainty and access to additional services.^8^ The most expansive undiagnosed initiative in the United States is the Undiagnosed Diseases Network (UDN), which encompasses 12 clinical sites and has, since its inception in 2014, cumulatively diagnosed over 400 individuals and described over 30 novel syndromes.^7^ Each UDN clinical site is staffed with specialists who develop and apply complex suites of bioinformatics tools to analyze sequencing data and uncover disease-causing variants.^9^ These sites each underwent a competitive application process and were selected to join the UDN due to their demonstrated track record of diagnosing difficult cases and characterizing novel genetic conditions through ongoing research efforts. The workflows implemented at these sites are thus representative of the state-of-the-art in rare disease diagnostic efforts.

We gathered details about 12 UDN bioinformatics pipelines, determined recurrent steps in a typical diagnostic evaluation, and identified consensus approaches. Moreover, we highlight substantial differences across pipelines regarding overall organization and incorporated tools. The comprehensive snapshot of effective computational workflows presented here can direct clinical teams interested in initiating genomic sequencing usage or re-evaluating patients who have had inconclusive genetic testing.

## MATERIALS AND METHODS

### Participating sites

Sequence analysis pipeline details were collected from the CLIA-certified sequencing core at Baylor Genetics (BaylorSeq) and 11 UDN clinical sites: Baylor College of Medicine (BCM), Duke University and Columbia University Institute for Genomic Medicine (Duke/Columbia), three Harvard-affiliated hospitals and Brigham Genomic Medicine (Harvard), University of Miami Miller School of Medicine (Miami), National Institutes of Health (NIH), University of Washington School of Medicine and Seattle Children’s Hospital (PacificNW), Stanford Center for Undiagnosed Diseases (Stanford), University of California–Los Angeles (UCLA), University of Utah Health Center for Genetic Discovery (Utah), Vanderbilt University Medical Center (Vanderbilt), and Washington University School of Medicine (WUSTL). The University of Pennsylvania and Children’s Hospital of Philadelphia clinical site had yet to process sequencing data for a UDN case at the time of writing and thus is excluded from this study.

### Data collection

We systematically collected details about each UDN site’s computational diagnostic workflows using a combination of in-person and virtual meetings with bioinformaticians and genetic counselors, online survey forms, and inspections of published papers and internal protocols.^10–12^

## RESULTS

### Overview of diagnostic workflow components

**Fig. 1:**
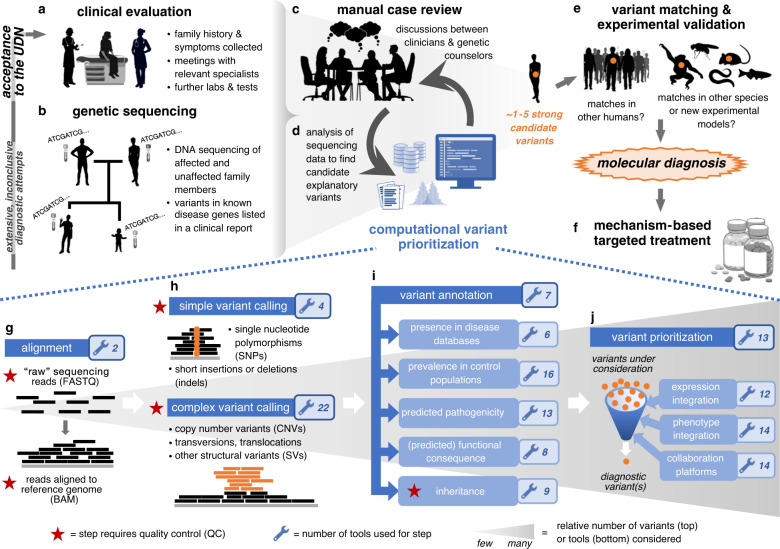
Representative clinical workflow to uncover disease-causing genetic variants in undiagnosed patients. Upon acceptance to the Undiagnosed Diseases Network (UDN), (**a**) an affected patient has an in-person clinical evaluation where extensive phenotyping and additional tests are performed as needed. (**b**) Before or during the clinical evaluation, samples of relevant affected and unaffected individuals in a family are sent for genomic sequencing. (**c**,**d**) Sequencing data provided by the sequencing center are analyzed in conjunction with other information in a back-and-forth process between bioinformaticians, clinicians, and genetic counselors to highlight variants that are likely to explain the patient’s disease. (**e**) Matches to the strong candidate explanatory variants identified in (**c**) are searched for in databases containing human genetic variant and corresponding symptom information (e.g., Matchmaker Exchange) or in databases containing animal genetic variants and corresponding phenotype information (e.g., MARRVEL). Strong candidate variants are also introduced into model organisms or cell lines where possible to assess in vivo phenotypic impact. (**f**) Once a candidate variant has been confirmed as disease causal, a molecular diagnosis is provided that can subsequently be used to tailor clinical management and molecular therapeutics. (**g**–**j**) Recurring steps in computational workflows to process genomic sequencing data to call, filter, and prioritize genetic variants that explain the affected individual’s disease symptoms.

Before applying to the UDN, a patient has typically endured extensive prior testing by multiple clinicians over the course of a multiyear “diagnostic odyssey.” As part of the application process, UDN clinical sites review patients’ health records to assess whether the UDN evaluation may aid in the identification of a diagnosis. Accepted patients undergo an in-person evaluation at a clinical site (Fig. 1a). In most cases, blood, saliva, and/or fibroblast samples of affected and unaffected individuals in the family are collected during this evaluation or beforehand via mailed-in collection kits. These samples are sequenced at BaylorSeq; all sequencing data are made available to the clinical site within weeks (Fig. 1b). Variants in disease-causing genes related to the clinical phenotype, medically actionable pathogenic variants in disease-causing genes unrelated to the clinical phenotype, and heterozygote status for select recessive Mendelian conditions are listed in a clinical report issued by BaylorSeq in accordance with the UDN protocol and following American College of Medical Genetics and Genomics (ACMG) variant classification guidelines.^13^ At 8 of the 11 clinical sites surveyed, researchers simultaneously perform local analyses of the sequencing data in an attempt to identify “strong candidate” variants that may explain the patient’s symptoms (Fig. 1c, d); three surveyed sites run their local pipelines only when BaylorSeq’s clinical report is inconclusive. Once candidate variants are highlighted via clinical sites’ and BaylorSeq’s analyses, there are three ways by which their causality is established. First, human and animal databases are queried for genotype-matched individuals with symptomatic concordance with the patient.^14–17^ Second, experiments are simultaneously performed to evaluate the in vivo effect of candidate variants in model organisms or cell lines. Third, the presence of secondary phenotypes indicated by genotype-matched individuals or in vivo experiments are confirmed in affected patients (Fig. 1e). Causal variants revealed through these steps are confirmed by Sanger sequencing, broadly shared by the UDN (Extended Data Note 1), and ideally lead to a molecular diagnosis for a patient, which in and of itself represents a turning point in a patient’s diagnostic odyssey, and also can inform positive therapeutic changes (Fig. 1f).^18^

The computational tools used to find explanatory genetic variants change constantly with newly available technologies and newly encountered disease etiologies. Despite these iterative improvements to bioinformatics pipelines, the primary roles that computational tools play in the overall variant prioritization process can be categorized as follows: (1) aligning sequencing reads to a reference human genome (Fig. 1g), (2) identifying genetic variants present in the individual from the sequencing reads (Fig. 1h), (3) annotating those variants with relevant information (Fig. 1i), and finally (4) filtering and prioritizing variants that are likely to cause the patient’s condition (Fig. 1j). In the following sections, we delve into the purpose of and tools used in each of these categories.

### Aligning next-generation sequencing reads

Aligning next-generation sequencing reads to a reference human genome is the necessary first step for all sequence analysis pipelines (Fig. 1g); the ubiquity of this step has resulted in community-driven standardization.^19^ Eight sites regularly realign reads after BaylorSeq’s initial alignment, whereas three sites realign reads only in specific circumstances, such as during reanalysis of a patient’s prior sequencing data. Realignment is necessary for six sites whose pipelines are configured for the GRCh37/hg19 human genome build, as genetic testing laboratories including BaylorSeq now provide reads aligned to the newer GRCh38/hg38 build. Realignment uses either an open-source implementation of the Burrows–Wheeler Aligner (BWA-MEM) (used regularly by six sites and in specific circumstances, as described above, by two sites) or Illumina/Edico’s DRAGEN aligner (used regularly by BaylorSeq and two clinical sites and in specific circumstances by one clinical site).

### Simple variant calling

Calling single-nucleotide variants (SNVs) and short insertions and deletions (indels) from aligned reads is the next step in sequence processing (Fig. 1h) and is often accomplished using the Genome Analysis Toolkit (GATK) best practices workflow,^20^ though Google’s DeepVariant^21^ and Real Time Genomics’ PolyBayes implementation (https://www.realtimegenomics.com) perform competitively for this task and are used in addition to GATK by two clinical sites. BaylorSeq calls variants using Illumina/Edico’s DRAGEN platform. Six clinical sites and BaylorSeq “jointly” call variants across samples as recommended in GATK to rescue low coverage true variants and accurately model false variants. In practice, variants are jointly called with (1) members of the same family, (2) other UDN patients at the same site, and/or (3) healthy patients internal or external to an institution. The Variant Quality Score Recalibration (VQSR) step recommended by GATK to identify technical artifacts, however, may misclassify real rare variants as false positives; this step is carefully reviewed or omitted in practice.

### Structural variant detection

In contrast to calling simple variants, calling structural variants (SVs) from GS data is a relatively divergent step, indicating that best practices have yet to be determined. SVs refer to large (>50 bp) insertions and deletions, duplications and other copy-number variants (CNVs), short tandem repeat (STR) expansions, translocations where genomic regions have moved within or across chromosomes, and inversions where a detached stretch of DNA was reattached in the opposite orientation. Combining the output from many SV calling tools—each optimized for detecting complementary types of SVs and often using distinct information (e.g., read depth, paired-end reads, or split reads)—is necessary for comprehensive SV detection.^22^ Existing SV detection tools have been reviewed in depth;^23^ here we list the subset of tools that are actively used by UDN sites (Table 1, Extended Data Table 1). The most commonly used tool, Manta, has been shown by independent evaluations to have high sensitivity but also a high false positive rate.^24^ Future development of SV benchmarking data sets for assessing the accuracy of SV detection tools will be essential in directing the current diverse exploration of techniques toward community-established best practices.

**Table 1:** Structural variant (SV) callers in use at clinical sites.

	BaylorSeq	BCM	Duke/Columbia	Harvard	Miami	NIH	PacificNW	Stanford	UCLA	Utah	Vanderbilt	WUSTL
**Find SVs from sequencing reads**												
Manta^a^	■	■	□	□	□	□	□	□	■	■	□	■
ExpansionHunter		■			■				■	■		■
GATK^b^		■		□		□			■			
LUMPY					□	■				□		□
CNVnator					□				■			■
RUFUS		■								■		
CNVkit								■				■
BreakDancer					□	■						
Illumina DRAGEN depth-based CNV caller	■											
SvABA: SV/indel Analysis by Assembly				■								
CoNIFER^c^							■					
ERDS: estimation by reads depth w/ SNVs									■			
BreakSeq2					□							
DELLY2					□							
**Jointly call and/or genotype SVs**												
smoove							■			■		■
SVTyper					□					□		□
**Annotate SVs**												
AnnotSV		■					■	■		■	■	■
gnomAD-SV					■							■
duphold										□		□
**Run or combine output from other tools**												
XHMM		■		■		■						
SURVIVOR					□							■
Parliament2					■							

■ Tool called directly. □ Tool called indirectly (e.g., by a wrapper).

Each SV calling tool identifies subsets of SVs by type or other factors, and so in practice, the output of multiple methods must typically be combined and considered together. Wrapper tools that automatically call and combine results from multiple other SV detection methods improve the efficiency of this process. Duke/Columbia, NIH, Stanford, and Vanderbilt only use SV calling tools in specific cases or contexts rather than as part of their regular pipelines. Tool citations are listed in Extended Data Table 1.

*CNV* copy-number variant, *SNV* single-nucleotide variant.

^a^Manta is used by BaylorSeq to generate putative SV calls, which are then shared with the clinical sites.

^b^The two functions from GATK used are GermlineCNVCaller and DepthOfCoverage (DoC); the latter is used to detect exonic deletions or duplications.

^c^In contrast to other tools, CoNIFER runs on exome sequencing (ES) data rather than genome sequencing (GS) data.

### Quality control of called variants

Confirming the quality of sequencing data and variants is critical to avoid expending downstream analyses on false variants. CLIA-certified genetic testing laboratories check the quality of unaligned and map-aligned sequencing reads prior to variant calling for all clinical grade sequencing (Extended Data Note 2). Four UDN clinical sites regularly confirm the quality of sequencing reads using a combination of FASTQC, FASTP, MultiQC, BEDTools (to check coverage), and bam.iobio. Other clinical sites begin quality control (QC) only after read alignment and variant calling.

QC for Mendelian disease diagnosis encompasses three checks: (1) sequencing reads are high quality, (2) sequenced samples correspond to the correct individuals and have expected relatedness, and (3) inheritance patterns across families are as expected (Table 2, Extended Data Table 1). BaylorSeq performs QC for all clinical genomic sequencing before providing data to UDN clinical sites. However, when patients provide their own sequencing data (as opposed to BaylorSeq providing newly acquired data) or when “research” (as opposed to clinical) sequencing is provided, clinical sites perform QC. Most sites have nearly identical steps for check 1 and similar QC for checks 2 and 3. In practice, QC has identified incorrectly related or labeled samples and poor overall quality of sequencing reads that were remedied via resequencing before subsequent analyses.^11^ Notably, existing QC tools rarely “flag” anomalous samples; users must accurately interpret results.

**Table 2:** Quality control (QC) checks of variants for rare disease diagnosis.

QC checks of variant data fall into three main categories, listed in bold above. Although some tools can be used for many of these steps, we illustrate here which QC steps they are actually used for in practice. Note the clarifications for some of the QC tools and steps listed in footnotes a–e. Tool citations are listed in Extended Data Table 1.

*ES* exome sequencing, *GS* genome sequencing, *SNV* single-nucleotide variant.

^a^BCFtools refers to the Wellcome Trust Sanger Institute’s suite of tools: BCFtools, VCFtools, SAMtools, and HTSlib.

^b^These tools either call *de novo* variants from sequencing reads to reduce false positive calls or provide *de novo* frequencies where a high frequency indicates a likely false positive.

^c^The expected transition (Ts) to transversion (Tv) ratios assume variants are called with respect to the human reference sequence; if variants are called with respect to computed ancestral alleles, the expected Ts/Tv ratio for ES should be ~1.

^d^Expected relatedness between family members is estimated using a “kinship coefficient”; unexpectedly low kinship implies a family member is not as related as was originally assumed, unexpectedly high kinship suggests consanguinity, and maximal kinship implies an accidental sample duplication.

^e^Mosaicism—where an individual contains a mix of genetically distinct cells—may be relevant for disease rather than only indicative of sequencing errors.

### Annotation and filtering of genetic variants

Even after removing low quality calls, a single genome can have several thousand unique genetic variants uncovered. Efficient, automated annotation and filtering of these variants is the next step of the variant prioritization process (Fig. 1i, Extended Data Table 2). Annotations fall into four categories: (1) known disease associations, (2) prevalence across healthy human populations, (3) predicted pathogenicity and functional effect, and (4) inheritance. Many scores exist across the first three categories;^25^ in the following sections we explore those that are used in practice for rare disease diagnosis.

### Known disease-associated genes

Many specific genetic variants have previously been determined to cause human disease, and it is useful to first look for the presence of these variants in a patient’s sequencing data. Databases compiling disease-causing variants, the genes they impact, and their phenotypic associations are used by ten clinical sites (Table 3). Genetic testing laboratories, including BaylorSeq, use these in addition to internal databases containing similar information. Disease-relevant variants are listed on clinical reports and are considered during the initial pass of each UDN case at all clinical sites.

**Table 3:** Human genetic variation data sets and derived tools.

	BaylorSeq	BCM	Duke/Columbia	Harvard	Miami	NIH	PacificNW	Stanford	UCLA	Utah	Vanderbilt	WUSTL
**Known disease gene databases**												
ClinVar	●	●	●	●	●	●	●	●	●	●	●	●
OMIM	●	●	●	●	●	●	●	●	●	●	●	●
HGMD: Human Gene Mutation Database	●	●	●	●		●			●	●	●	
dbSNP	●	●	●				●			●		
CGD: Clinical Genomic Database										●	●	
Orphanet								●			●	
**Healthy human population single-nucleotide variant (SNV)/indel databases**												
gnomAD: Genome Aggregation Database	●	●	●	●	●	●	●	●	●	●	●	●
ExAC: Exome Aggregation Consortium	●	●	●	●	●		●	●	●		⚬	●
1000 Genomes Project	●		●				●	●	●	●	●	●
Institution—internal controls^a^		●	●	●		●	●		●	●	●	
EVS: Exome Variant Server	●		●		●		●	●				
TOPMed: Trans-Omics for Precision Medicine			⚬				●	●		⚬		⚬
UK10K							●	●	●			
Greater Middle East (GME) Variome Project			⚬									⚬
xKJPN: 1000+ Japanese			⚬									
GenomeAsia 100 K Project			⚬									
Iranome			⚬									
**Human structural variant (SV) databases**												
gnomAD-SV: Genome Aggregation Database SVs		●		⚬	●		●	●	●	●	●	●
DGV: Database of Genomic Variants		●		⚬			●	●	●	●	●	●
dbVar: Database of Genomic Structural Variation		●						●		●		●
ClinGen: Clinical Genome Resource		●		⚬				●		●		●
DECIPHER		●		⚬			●			●		
Institution—internal controls^a^									●	●		●
**Within-human selective constraint scores**												
pLI: probability of loss-of-function (LoF) intolerance		●	●	●	●	●		⚬	●	●	●	●
Missense (constraint) *Z* score		●	●		●	●	●		●	●		
pREC: probability of homozygote LoF intolerance			●	⚬					●			
(sub)RVIS: Residual Variation Intolerance Score			●			●						
L-o/e-UF: LoF observed/expected upper-bound fraction				●			●					
CCR: constrained coding regions									●	●		
LIMBR: Localized Intolerance Model w/ Bayesian Regression			●									
MTR: missense tolerance ratio			●									
s_het: selective effect of heterozygous LoF				●								
M-o/e-UF: missense observed/expected upper-bound fraction							●					
LoFtool										●		
● Tool used by default. ⚬ Tool used in specific cases or contexts only.^b^												

Knowledge of variation within human populations with and without disease can be effectively used to assess the likelihood of a variant to cause the genetic condition under investigation. Tool and data set citations are listed in Extended Data Table 1.

^a^Human sequence variation data sets that are internal to particular institutions and used by clinical sites surveyed here include variants present in patients from Baylor College of Medicine (BCM), the Institute for Genomic Medicine (Duke/Columbia), Brigham Genomic Medicine (Harvard), the NIH Undiagnosed Diseases Program (NIH), Centers for Mendelian Genomics (PacificNW), University of California–Los Angeles (UCLA), the Centre d’Etude du Polymorphisme Humain (Utah), and BioVu (Vanderbilt), and a curated set of copy-number variants (CNVs) detected via genome sequencing (GS) and confirmed via chromosomal microarray analysis (Washington University School of Medicine [WUSTL]).

^b^The contexts in which specific human population variant data sets are used include historical reasons (ExAC), when a variant’s gnomAD-derived MAF is 0 or close to 0 (TOPMed), when patients’ inferred ancestry is non-European (TOPMed), Middle Eastern (GME), Japanese (xKJPN), Asian (GenomeAsia), and/or Iranian (Iranome), and when a predicted structural variant impacts a clinically relevant gene (gnomAD-SV, DGV, ClinGen, DECIPHER).

### Variant segregation in healthy human populations

Several positions within the human genome naturally vary across healthy individuals, and “common” variants at these positions are unlikely to cause the conditions under investigation by the UDN. Though rare combinations of otherwise common variants may lead to disease,^26^ clinical sites do not currently consider all common variant combinations. Instead, variants observed more than 1 in 100 times across healthy populations (i.e., minor allele frequency [MAF] > 0.01) are typically excluded during the first pass of the data. The exact MAF threshold used depends on the suspected mode of inheritance. Lower MAF thresholds are used for suspected dominant conditions because the variants causing the extremely rare phenotypes of UDN patients are assumed to be naturally selected against and thus equally rare in the general population and entirely absent in control population databases. Higher MAF thresholds are used for suspected recessive conditions because heterozygous individuals would not be expected to manifest severe disease features.

All UDN sites use data from the Broad Institute’s Genome Aggregation Database (gnomAD) to compute MAFs, and seven sites also compute MAFs from smaller or population-specific data sets on a case-by-case basis (Table 3). Two sites eliminate variants that are homozygous in three or more healthy individuals in these data sets. At the NIH site, rather than thresholding on MAFs computed directly from variant proportions in gnomAD, 95% Wilson confidence score intervals computed from these proportions are used to retain rare variants occurring in low coverage regions. Finally, five sites flag variants that are present in data sets internal to their institutions, because variants present in asymptomatic or differently symptomatic individuals are unlikely to be disease-relevant.

Eight sites consult SV databases to check the existence and/or MAF of detected SVs (Table 3, Extended Data Table 1). Multiple databases are checked in practice because the SV detection tools used across databases differ, so the absence or rarity of an SV in one database may reflect a particular SV detection approach rather than true population rarity.

Simple genetic variation observed across healthy humans tends to be sparsely distributed with varying degrees of impact. These features can be used to capture how regions of the human genome may be intolerant of loss-of-function (LoF) variants, such as frameshift or protein-truncating variants. Nine surveyed sites incorporate selective constraint scores derived from and released with gnomAD data in their diagnostic pipelines, with the probability of heterozygous LoF intolerance scores and missense constraint *Z* scores used most commonly (Table 3).

### Predicted pathogenicity and functional effect of variants

Various tools predict the pathogenicity of uncovered variants.^25^ Values derived from cross-species comparative genomics contribute heavily to pathogenicity predictors, as positions that are conserved across species tend to be functionally critical. However, since most candidate coding variants are evolutionarily well-conserved, only five sites directly consider conservation in their diagnostic pipelines (Table 4, Extended Data Table 1).

**Table 4:** Tools for assigning the pathogenic likelihood or functional impact of variants.

	BaylorSeq	BCM	Duke/Columbia	Harvard	Miami	NIH	PacificNW	Stanford	UCLA	Utah	Vanderbilt	WUSTL
**Cross-species conservation scores**												
GERP++: Genomic Evolutionary Rate Profiling		●	●			●	●				●	
PhastCons					●	●	●					
**Predicted functionality or pathogenicity**												
PolyPhen-2	●	●	●	●	●	●	●	●	●	●		
SIFT	●	●		●	●	●	●	●	●	●		
MutationTaster				●	●	●		●				
MVP: missense variant pathogenicity								●				
ReMM: regulatory Mendelian mutation								●				
**Ensemble pathogenicity predictors**												
CADD: Combined Annotation Dependent Depletion	●	●			●	●	●	●			●	●
REVEL: Rare Exome Variant Ensemble Learner		●	●	●		●	●	●		●		●
DANN: Deep Neural Net version of CADD	●				●							
M-CAP: Mendelian Clinically Applicable Pathogenicity								●				●
DOMINO: Dominant Disorder Associated Genes^a^		●										
Eigen						●						
**Predicted splice- or expression-altering effect**												
SpliceAI	●	●		●	●		●	●	●	●	●	●
GTEx: Genotype-Tissue Expression		●			●			●		●		
SpliceRegion annotations from VEP							●	●		●		●
dbscSNV (splicing consensus SNVs)					●				●			●
Human Splicing Factor		●									●	
MMSplice: Modular modeling of splicing					●	●						
MaxEntScan						●					●	
TraP: Transcript-inferred Pathogenicity			●									

Variants of uncertain significance (i.e., that are not already known to be associated with disease) can be evaluated for functional or pathogenic impact using predictive models. Tool citations are listed in Extended Data Table 1.

^a^Unlike other tools, DOMINO provides scores per gene rather than per variant.

The most commonly used pathogenicity predictors for rare disease diagnosis—used by eight clinical sites each—are Combined Annotation Dependent Depletion (CADD) and Rare Exome Variant Ensemble Learner (REVEL), each of which consider multiple variant annotations and where scores >25 and >0.3 respectively indicate likely pathogenic variants. Nearly all predicted pathogenicity scores used, with the exception of ReMM, indicate disease relevance primarily for coding variants.^27^

Indeed, predicting and experimentally validating the pathogenic impact of noncoding variants is notoriously difficult. All 12 sites use tools to predict how noncoding variants alter expected gene expression and splicing. Few sites use the same subset of tools for this task, though SpliceAI is the most commonly used tool overall (Table 4).

### Mode of inheritance

After variants have been quality checked, MAF filtered, and annotated, Mendelian mode of inheritance is evaluated next by the clinical sites. Some sites simultaneously consider the functional impact of variants, where, for instance, intergenic or perceived synonymous variants are excluded.^3^ Despite the ubiquity of this step, each site uses different tools for computing inheritance patterns.

For a dominantly inherited genetic condition to manifest, only one defective copy of the relevant gene is required, whereas recessive disease manifestation requires two defective gene copies. GS of unrelated or distantly related affected individuals is desired in suspected dominant cases to find rare, shared variants.

In sporadic cases—caused by a single *de novo* dominant or two recessive variants—GS of at least the affected individual and both unaffected parents is desired. Selecting heterozygous variants in the affected individual that are absent in both unaffected parents or homozygous variants in the affected individual that are absent in at least one parent via straightforward segregation analysis results in a majority of spurious *de novo* calls. These false positive calls stem from inadequate sequence coverage or alignment in parents from whom variants were in fact inherited and/or inaccurate modeling of underlying variant frequencies. Four sites regularly use specialized *de novo* calling tools or databases to offset these issues (Table 2). Fixing *de novo* calling errors requires analysis of sequencing reads, which many genetic testing centers do not readily provide.

Occasionally in sporadic and/or recessive cases, the same disease-causing variant is inherited from both heterozygous parents and can be easily detected as a homozygous variant. Genomic regions containing only homozygous variants in an affected individual with nonconsanguineous parents can also indicate an inherited deletion from one parent or uniparental isodisomy. These latter phenomena, revealed as Mendelian violations during the QC process (Table 2), can manifest in a recessive disease despite only one parent being heterozygous for the disease-causing variant. Often in undiagnosed recessive cases, two or more different heterozygous variants, each either inherited or occurring *de novo*, can give rise to the disease phenotype; these variants are referred to as compound heterozygous pairs. The complete set of compound heterozygous variant pairs in any given case is very large, and so filters—such as restricting to rare, LoF, likely pathogenic variants—are applied beforehand. If too few candidate explanatory variants pass these filters, the NIH, WUSTL and Miami sites use internal “second tier” schemes, such as increasing the allowable MAF threshold, to rescue additional compound heterozygous pairs.^28^

### Integration of nonsequencing data

Cases with nondiagnostic genetic testing have eventually been solved by reanalysis approaches that leverage additional data, such as transcriptome sequencing^29,30^ (RNA-seq) or “deep phenotyping,”^31,32^ to complement ES and GS.

### Transcriptome sequencing

RNA-seq is increasingly utilized to (1) confirm suspected expression- or splice-altering variants initially prioritized through genomic sequencing, and/or (2) highlight genes that are aberrantly expressed relative to healthy, tissue-matched samples from databases such as GTEx (https://gtexportal.org/).^29,30^ BCM, Stanford, and UCLA regularly use RNA-seq data for variant prioritization, and two other sites are actively working to incorporate RNA-seq data into their workflows as well (Extended Data Table 3). Vanderbilt uses PrediXcan to correlate observed phenotypes with imputed, rather than directly measured, gene expression.^33^

### Structured phenotyping

Deep phenotyping of patients is critical to the overall UDN process (Fig. 1a) and enables clinicians to focus on genes associated with a patient’s symptoms or suspected disease. Symptom terms are standardized via the Human Phenotype Ontology (HPO) and explicitly annotated for each UDN case during the in-person evaluation.^34^ Computational tools can reason over these terms to generate gene panels that complement manual efforts.^35^ All clinical sites have access to genes ranked by PhenoTips, a program embedded into the UDN data server. Eight clinical sites and BaylorSeq use additional tools to prioritize genes from patients’ phenotypes (Fig. 1j, Extended Data Table 4).^36^ Amelie is used by five sites to scour the literature for examples of genes causing patients’ observed phenotypes, a process typically performed manually using the Monarch Initiative’s gene–phenotype browser. Exomiser is used by three sites to integrate genotype–phenotype data and runs in parallel to existing pipelines. Finally, pairwise associations between genes and HPO terms are downloadable from the HPO website; the union of genes associated with all annotated HPO terms per patient can be used directly or intersected with sets of disease-relevant genes from OMIM and HGMD. This approach is used by three sites regularly but has been implemented for various projects at all clinical sites.

### Workflow management and wrapper tools

The complex workflows described here must be well-documented, customizable per case, and provide results in a timely manner and intuitive format. Case materials should be accessible by collaborative teams of clinicians, bioinformaticians, and genetic counselors. In practice, all sites use automated platforms to call, annotate, and prioritize candidate diagnostic variants (Extended Data Table 5, Extended Data Table 6). Spreadsheets are the most common tool used by all sites for storing, sharing, and commenting on variant-level data. Many sites also use commercial solutions for case management, which has enabled secure transition of certain workflow components to the cloud.

## DISCUSSION

Pinpointing the genetic variants giving rise to ultrarare, undiagnosed diseases is a challenging and pressing problem being tackled on a case-by-case basis by clinical researchers worldwide. The computational tools utilized during these investigative efforts reflect relevant community standards but can also diverge across institutions and even across cases handled by the same clinical team.

The diverse, exploratory techniques employed by UDN clinical sites can overcome inherent limitations of clinical case review and standard sequencing interpretation provided by genetic testing laboratories—both of which rely on existing disease gene knowledge—by uncovering novel disease loci. For instance, when no compelling variants were found in phenotypically prioritized genes in two patients presenting with muscular and white matter abnormalities, a genetics-driven UDN pipeline uncovered diagnostic *de novo* missense variants in both individuals in *TOMM70*, a gene previously unassociated with disease.^37^ Similarly, sequencing analyses were able to uncover *de novo*, heterozygous variants in nine individuals with neurodevelopmental delay and other multisystem anomalies in *CDH2*, a gene previously unassociated with a Mendelian neurodevelopmental condition.^38^

Indeed, divergent aspects of UDN pipelines reflect promising avenues for case reanalysis and reveal areas where technical developments would be most impactful. Improving SV detection specificity would aid in cases with nondiagnostic microarrays, gene panels, and GS. Experimentally verifiable pathogenicity predictions for noncoding variants may solve cases with nondiagnostic ES. Finally, automated integration of additional data, such as RNA-seq,^29,30^ long-read sequencing,^39^ and epigenetic modifications,^40^ may also increase the diagnostic rate for cases with inconclusive GS.

Consensus tools used across sites by multiple clinical research teams have been convincingly evaluated and are easily incorporated into existing workflows external to their original development environment. Clinical sites strive to incorporate better tools—including those developed in-house—as they emerge over time. Flexible, open-source implementations ease this process and can ultimately shorten the time to and improve the rate of diagnosis. Initiatives like the UDN provide an excellent opportunity to assess and share tools and ideas and jointly develop methods inspired by the most challenging undiagnosed cases.

## Supplementary information

Extended Data

## Data Availability

All data used in this analysis are available in the Main and Extended Data Tables.
