# Supplementary material for: Commonalities across computational workflows for uncovering explanatory variants in undiagnosed cases
Source: Genet Med. 2021 Feb 12;23(6):1075–85. doi: 10.1038/s41436-020-01084-8 (PMC8187147; doi:10.1038/s41436-020-01084-8)
Supplement: Supplementary file 1 — Extended Data [file 41436_2020_1084_MOESM1_ESM.pdf]

Extended Data

Extended Data Note 1. Sharing candidate etiological variants in UDN cases.

Strong candidate or confirmed diagnostic variants uncovered across all UDN clinical sites are submitted to ClinVar every three to four months by the UDN Coordinating Center (https://www.ncbi.nlm.nih.gov/clinvar/submitters/505999/). Cases with strong candidate variants are also shared via Matchmaker Exchange through the PhenomeCentral node (https://www.phenomecentral.org). In addition, patients have the option of listing their candidate gene/variant data on the public-facing UDN website (https://undiagnosed.hms.harvard.edu/genes/). Finally, strong candidate genes are listed on a gene function study candidate list for further investigation (https://undiagnosed.hms.harvard.edu/research/funding-opportunities/).

Extended Data Note 2. Baylor Genetics' sequencing coverage and sample identity checks.

Depth of sequencing coverage across targeted regions is checked by all CLIA-certified genetic testing laboratories including the Baylor Genetics sequencing core before sequencing data is made available to a client. The most recent coverage requirements (as of September 2020) for clinical genomic sequencing by Baylor Genetics are: average coverage over the genome must be >40X, and >97.5% of the "target base" (i.e., all coding regions, UTRs, noncoding genes and intronic regions implicated in human disease) must be covered at >20X.

In addition, as a quality control measure for each sample, concordance between variants called from next-generation sequencing (NGS) data and 96 SNPs measured by an independent genotype array must be >95%. Specifically, the genotype array is performed by the Fluidigm SNPtype platform using the SNPTrace Panel, which consists of 46 autosomal SNPs that are polymorphic across all populations, 44 autosomal ancestry-informative SNPs, 3 SNPs on chromosome X, and 3 SNPs on chromosome Y. The SNP data are compared with the genotype calls made from the Illumina Dragen BioIT Platform for NGS data to ensure correct sample identification. Contamination analysis is also performed by identifying homozygous sites and by computationally inspecting the aligned reads at those sites.

**Main Text**	
▸ **Aligning next-generation sequencing reads**	Burrows-Wheeler Aligner (BWA-MEM)[1], Illumina/Edico's DRAGEN aligner (https://www.illumina.com/products/by-type/informatics-products/dragen-bio-it-platform.html)
▸ **SNP/indel variant calling**	GATK[2], DeepVariant[3], Real Time Genomics' PolyBayes (https://www.realtimegenomics.com)[4]
▸ **Quality control of sequencing reads**	FASTQC (https://www.bioinformatics.babraham.ac.uk/projects/fastqc/), FASTP[5], MultiQC[6], BEDTools[7], bam.iobio[8]
▸ **Patient–gene matching (Figure 1)**	Matchmaker Exchange[9], MARRVEL[10]
**Table 1**	
▸ **Find SVs from sequencing reads**	Manta[11], ExpansionHunter[12], GATK[2], LUMPY[13], CNVnator[14], CNVkit[15], RUFUS (https://github.com/jandrewrfarrell/rufus), BreakDancer[16], SvABA[17], CoNIFER[18], ERDS[19], BreakSeq2[20], DELLY2[21]
▸ **Jointly call and/or genotype SVs**	smoove (https://github.com/brentp/smoove), SVTyper[22]
▸ **Annotate SVs**	AnnotSV[23], gnomAD-SV[24], duphold[25]
▸ **Run or combine output from other tools**	XHMM[26], SURVIVOR[27], Parliament2[28]
**Table 2**	
	BCFtools[29], vcf.iobio (http://vcf.iobio.io), GATK's DepthOfCoverage[2], Integrative Genomics Viewer (IGV)[30], Peddy[31], LAMP-LD[32], plink[33], ATAV (https://github.com/igm-team/atav), denovo-db (https://denovo-db.gs.washington.edu), NovoCaller[34], Salvage Pathway[35]
**Table 3**	
▸ **Known disease gene databases**	ClinVar[36] and ClinVar Miner[37], OMIM[38], HGMD[39], dbSNP[40], CGD[41], Orphanet (https://www.orpha.net/)
▸ **Healthy human population SNV/indel databases**	gnomAD[42], ExAC[43], 1000 Genomes Project[44], EVS[45], TOPMed (https://www.nhlbiwgs.org), UK10K[46], GME[47], xKJPN[48], GenomeAsia 100K Project[49], Iranome[50]
▸ **Human structural variant databases**	gnomAD-SV[24], DGV[51], dbVar[52], ClinGen's Dosage Sensitivity Map (https://dosage.clinicalgenome.org), DECIPHER[53]
▸ **Within-human selective constraint scores**	pLI[43], missense (constraint) *Z* score[43], pREC[43], RVIS[54,55], subRVIS[54,55], LoF/missense-o/e-UF[42], CCR[56], LIMBR[57], MTR[58], s_het[59], LoFtool[60]
**Table 4**	
▸ **Cross-species conservation scores**	GERP++[61], PhastCons[62]
▸ **Predicted functionality or pathogenicity**	PolyPhen2[63], SIFT[64], MutationTaster[65], MVP (https://github.com/ShenLab/missense), ReMM[66]
▸ **Ensemble pathogenicity predictors**	CADD[67], REVEL[68], DANN[69], M-CAP[70], DOMINO[71], Eigen[72]
▸ **Predicted splice- or expression-altering effect**	SpliceAI[73], GTEx (https://www.gtexportal.org), SpliceRegion[74], dbscSNV[75], Human Splicing Factor[76], MMSplice[77], MaxEntScan[78], TraP[79]

Extended Data Table 1. Citations of tools listed in main text and Tables 1–4.

References for computational approaches and datasets listed in Tables 1-4; links are provided for open-source tools that are currently unpublished.

Variant annotation tools	BaylorSeq	BCM	Duke/Columbia	Harvard	Miami	NIH	PacificNW	Stanford	UCLA	Utah	Vanderbilt	WUSTL
Ensembl's Variant Effect Predictor (VEP)[80]				●	●		●			●		○
Annovar[81]		●				●						●
SnpEff/ClinEff[82]			●							○		
GenomOncology Knowledge Management API[83]	●											
Interactive Biosoftware’s commercial Alamut (https://www.interactive-biosoftware.com)	○											
GoldenHelix's commercial VarSeq[84]									●			
SeattleSeq Annotation Server[85]							●					

● = *tool used by default* ○ = *tool used in specific cases or contexts only*[a]

Extended Data Table 2. Annotating SNV and indel variants.

Tools for annotating genetic variants with their functional effects and relevant additional information. Clinical sites listed in gray do not separately annotate simple variants; this is accomplished using an automated wrapper tool (i.e., Exomiser at Stanford and emedgene at Vanderbilt). [a]The specific contexts in which some tools are used include: to consider splice region annotations specifically (Ensembl VEP) and to verify selected variants where there is uncertainty from default annotation tools (SnpEff/ClinEff and Alamut).

Tool Name	Tool Purpose	BCM	NIH	Stanford	UCLA	Utah
Trimmomatic[86]	preprocess raw reads		●			
Cutadapt[87]	〃			●		
STAR[88]	align reads	●	●	●	●	●
featureCounts[89]	measure read depth per exon/gene		●		●	
RSEM[90]	〃	●		●		
DESeq2[91]	analyze differential expression		●			●
edgeR[92]	〃		●			
OUTRIDER[93]	detect expression anomalies	●	△		●	
custom scripts	〃			●	●	
LeafCutter[94]	detect splice junction distributions and anomalies		△			
RIVER[95]	〃			●		
FRASER*	〃	●				
custom scripts	〃			●	●	

● = *tool used by default* △ = *tool has been tested*

Extended Data Table 3. Processing transcriptomic data.

Tools actively used or being tested by clinical sites for processing RNA-sequencing data. *FRASER is unpublished but available at https://github.com/c-mertes/FRASER and in practice is called via DROP (Detection of RNA Outlier Pipeline), a wrapper tool available at https://github.com/gagneurlab/drop.

	BaylorSeq	BCM	Duke/Columbia	Harvard	Miami	NIH	PacificNW	Stanford	UCLA	Utah	Vanderbilt	WUSTL
**Variant prioritization tools based on phenotype and genotype input**												
Amelie[96]						○		○		●	○	○
Exomiser[97]				○		●		●				
emedgene (https://emedgene.com)		●									●	
OMIM Explorer[98]	●											
Codified (https://www.codifiedgenomics.com)		●										
GENESIS[99]					●							
**Gene prioritization tools based only on phenotype input**												
PhenoTips[100]							●	●	●			
Phenomizer[101]							●					
genepanel.iobio[102]										●		
→ NCBI's Genetic Testing Registry (GTR)[103]										○		
→ Phenolyzer[104]										○		
**Databases with parseable gene–phenotype relationships**												
Monarch Initiative's Gene/PubMed Browser[105]							●			●		
Mouse Genome Informatics (MGI)[106]		●	○						●			
HPO–gene annotations[107]	○								●	○		

● = *tool used by default* ○ = *tool used in specific cases or contexts only*[a]

Extended Data Table 4. Incorporating phenotype data in the gene- and variant-prioritization process.

Computational tools take phenotype information in the form of structured Human Phenotype Ontology (HPO) terms in order to prioritize phenotypically-relevant variants and genes. Clinical sites that do not regularly use phenotype information in their automated pipelines are listed in gray in the top row. The "→" indicates tools that are not used directly but rather are accessed indirectly by the wrapper tool genepanel.iobio. [a]The specific contexts in which some tools are used include: to find supporting literature when the molecular mechanism of a candidate variant is unclear (Amelie), to investigate phenotypically-ranked variants in cases suggestive of a known disease (Exomiser), to confirm the validity of genepanel.iobio's suggested gene lists (GTR, Phenolyzer), when particular genetic counselors are assigned to a case (MGI), and when more exhaustive lists of phenotypically-associated genes are desired (HPO–gene annotations).

	Variant Filtering	Pipeline Automation	Case Collaboration	Custom Programs	BaylorSeq	BCM	Duke/Columbia	Harvard	Miami	NIH	PacificNW	Stanford	UCLA	Utah	Vanderbilt	WUSTL
MS Office365 Excel spreadsheets	✓		✓		●	●	●	●	●		●	●	●	○	●	●
Box/Dropbox			✓			●		●	●		●	●	●	●	●	
GitHub				✓			●			●	●	●	●	●		
Custom Laboratory Information Management System (LIMS)[108]			✓		●	●				●	●					
Exomiser[109]	✓	✓						○		●		●				
MS Office365 SharePoint		✓	✓	✓		●					●					
emedgene	✓	✓				●									●	
xBrowse/seqr	✓	✓	✓				●	●								
REDCap[110]			✓						●		●					
GEMINI[111]	✓	✓								●	●					
Codified	✓	✓				●										
Redmine		✓	✓	✓			●									
ATAV	✓	✓					●									
MS Office365 OneNote			✓				●									
Forome Anfisa[112]	✓	✓	✓					○								
GENESIS[99]	✓	✓	✓						●							
Snakemake		✓		✓								●				
DNAnexus		✓		✓									●			
VarSeq	✓	✓	✓	✓									●			
Jira		✓	✓	✓										●		
slivar	✓													●		
frameshift.io (mosaic)	✓		✓											●		
Genome Modeling System[113]		✓	✓	✓												●

● = *tool used by default* ○ = *tool used in specific cases or contexts only*[a]

Extended Data Table 5. Case workflow management and collaboration tools.

Distinct sets of tools or platforms are used to automate the case processing and variant prioritization workflows and to enable clinical collaboration at each UDN clinical site. The majority of UDN sites use commercially available software or tools for pipeline management. Current web links to tools listed in this table can be found in Extended Data Table 6. [a]The specific contexts in which some tools are used include: to share variant-level data as requested with clinicians or genetic counselors (MS Office365 Excel spreadsheets), to prioritize phenotypically-relevant variants in cases suggestive of known diseases (Exomiser), and to consider newer or additional annotations (Forome Anfisa).

Tool Name	Availability
MS Office365 Excel	https://www.microsoft.com/en-us/microsoft-365/excel
Box	https://www.box.com/
Dropbox	https://www.dropbox.com
GitHub	https://github.com
Exomiser[109]	https://github.com/exomiser/Exomiser
MS Office365 SharePoint	https://www.microsoft.com/en-us/microsoft-365/sharepoint
emedgene	https://emedgene.com
xBrowse/seqr	https://seqr.broadinstitute.org
REDCap[110]	https://www.project-redcap.org
GEMINI[111]	https://gemini.readthedocs.io/en/latest/
Codified	http://codifiedgenomics.com/
Redmine	https://www.redmine.org
ATAV	https://github.com/igm-team/atav
MS Office365 OneNote	https://www.microsoft.com/en-us/microsoft-365/onenote
Forome Anfisa[112]	https://forome.org
GENESIS[99]	https://genesis-app.com
Snakemake	https://snakemake.readthedocs.io/en/stable/
DNAnexus	https://www.dnanexus.com
VarSeq[84]	https://www.goldenhelix.com/products/VarSeq/
Jira	https://www.atlassian.com/software/jira
slivar	https://github.com/brentp/slivar/wiki/rare-disease
frameshift.io (mosaic)	https://frameshift.io/mosaic/
Genome Modeling System[113]	https://github.com/genome/gms

Extended Data Table 6. Access to workflow management and collaboration tools.

Case workflow management and collaboration tools used by UDN clinical sites are either open-source or commercially-available.

Extended Data References

1. Li, H. & Durbin, R. Fast and accurate short read alignment with Burrows-Wheeler transform. *Bioinformatics* **25**, 1754–1760 (2009).
2. Van der Auwera, G. A. *et al.* From FastQ data to high confidence variant calls: the Genome Analysis Toolkit best practices pipeline. *Curr. Protoc. Bioinformatics* **43**, 11.10.1–11.10.33 (2013).
3. Poplin, R. *et al.* A universal SNP and small-indel variant caller using deep neural networks. *Nat. Biotechnol.* **36**, 983–987 (2018).
4. Marth, G. T. *et al.* A general approach to single-nucleotide polymorphism discovery. *Nat. Genet.* **23**, 452–456 (1999).
5. Chen, S., Zhou, Y., Chen, Y. & Gu, J. fastp: an ultra-fast all-in-one FASTQ preprocessor. *Bioinformatics* **34**, i884–i890 (2018).
6. Ewels, P., Magnusson, M., Lundin, S. & Käller, M. MultiQC: summarize analysis results for multiple tools and samples in a single report. *Bioinformatics* **32**, 3047–3048 (2016).
7. Quinlan, A. R. & Hall, I. M. BEDTools: a flexible suite of utilities for comparing genomic features. *Bioinformatics* **26**, 841–842 (2010).
8. Miller, C. A., Qiao, Y., DiSera, T., D'Astous, B. & Marth, G. T. bam.iobio: a web-based, real-time, sequence alignment file inspector. *Nat. Methods* **11**, 1189 (2014).
9. Philippakis, A. A. *et al.* The Matchmaker Exchange: a platform for rare disease gene discovery. *Hum. Mutat.* **36**, 915–921 (2015).
10. Wang, J. *et al.* MARRVEL: Integration of Human and Model Organism Genetic Resources to Facilitate Functional Annotation of the Human Genome. *Am. J. Hum. Genet.* **100**, 843–853 (2017).
11. Chen, X. *et al.* Manta: rapid detection of structural variants and indels for germline and cancer sequencing applications. *Bioinformatics* **32**, 1220–1222 (2016).
12. Dolzhenko, E. *et al.* ExpansionHunter: a sequence-graph-based tool to analyze variation in short tandem repeat regions. *Bioinformatics* **35**, 4754–4756 (2019).
13. Layer, R. M., Chiang, C., Quinlan, A. R. & Hall, I. M. LUMPY: a probabilistic framework for structural variant discovery. *Genome Biol.* **15**, R84 (2014).
14. Abyzov, A., Urban, A. E., Snyder, M. & Gerstein, M. CNVnator: an approach to discover, genotype, and characterize typical and atypical CNVs from family and population genome sequencing. *Genome Res.* **21**, 974–984 (2011).
15. Talevich, E., Shain, A. H., Botton, T. & Bastian, B. C. CNVkit: Genome-Wide Copy Number Detection and Visualization from Targeted DNA Sequencing. *PLoS Comput. Biol.* **12**, e1004873 (2016).
16. Chen, K. *et al.* BreakDancer: an algorithm for high-resolution mapping of genomic structural variation. *Nat. Methods* **6**, 677–681 (2009).
17. Wala, J. A. *et al.* SvABA: genome-wide detection of structural variants and indels by local assembly. *Genome Res.* **28**, 581–591 (2018).
18. Krumm, N. *et al.* Copy number variation detection and genotyping from exome sequence data. *Genome Res.* **22**, 1525–1532 (2012).
19. Zhu, M. *et al.* Using ERDS to infer copy-number variants in high-coverage genomes. *Am. J. Hum. Genet.* **91**, 408–421 (2012).
20. Lam, H. Y. K. *et al.* Nucleotide-resolution analysis of structural variants using BreakSeq and a breakpoint library. *Nat. Biotechnol.* **28**, 47–55 (2010).
21. Rausch, T. *et al.* DELLY: structural variant discovery by integrated paired-end and split-read analysis. *Bioinformatics* **28**, i333–i339 (2012).
22. Chiang, C. *et al.* SpeedSeq: ultra-fast personal genome analysis and interpretation. *Nat. Methods* **12**, 966–968 (2015).
23. Geoffroy, V. *et al.* AnnotSV: an integrated tool for structural variations annotation. *Bioinformatics* **34**, 3572–3574 (2018).

24. Collins, R. L. *et al.* A structural variation reference for medical and population genetics. *Nature* **581**, 444–451 (2020).
25. Pedersen, B. S. & Quinlan, A. R. Duphold: scalable, depth-based annotation and curation of high-confidence structural variant calls. *Gigascience* **8**, (2019).
26. Fromer, M. & Purcell, S. M. Using XHMM Software to Detect Copy Number Variation in Whole-Exome Sequencing Data. *Curr. Protoc. Hum. Genet.* **81**, 7.23.1–21 (2014).
27. Jeffares, D. C. *et al.* Transient structural variations have strong effects on quantitative traits and reproductive isolation in fission yeast. *Nat. Commun.* **8**, 14061 (2017).
28. Zarate, S. *et al.* Parliament2: Fast Structural Variant Calling Using Optimized Combinations of Callers. doi:10.1101/424267.
29. Li, H. *et al.* The Sequence Alignment/Map format and SAMtools. *Bioinformatics* **25**, 2078–2079 (2009).
30. Robinson, J. T. *et al.* Integrative genomics viewer. *Nat. Biotechnol.* **29**, 24–26 (2011).
31. Pedersen, B. S. & Quinlan, A. R. Who's Who? Detecting and Resolving Sample Anomalies in Human DNA Sequencing Studies with Peddy. *Am. J. Hum. Genet.* **100**, 406–413 (2017).
32. Baran, Y. *et al.* Fast and accurate inference of local ancestry in Latino populations. *Bioinformatics* **28**, 1359–1367 (2012).
33. Purcell, S. *et al.* PLINK: a tool set for whole-genome association and population-based linkage analyses. *Am. J. Hum. Genet.* **81**, 559–575 (2007).
34. Mohanty, A. K. *et al.* novoCaller: a Bayesian network approach for de novo variant calling from pedigree and population sequence data. *Bioinformatics* **35**, 1174–1180 (2019).
35. Gu, F. *et al.* A suite of automated sequence analyses reduces the number of candidate deleterious variants and reveals a difference between probands and unaffected siblings. *Genet. Med.* **21**, 1772–1780 (2019).
36. Landrum, M. J. *et al.* ClinVar: improving access to variant interpretations and supporting evidence. *Nucleic Acids Res.* **46**, D1062–D1067 (2018).
37. Henrie, A. *et al.* ClinVar Miner: Demonstrating utility of a Web-based tool for viewing and filtering ClinVar data. *Hum. Mutat.* **39**, 1051–1060 (2018).
38. OMIM (TM). *SpringerReference* doi:10.1007/springerreference_36173.
39. Stenson, P. D. *et al.* Human Gene Mutation Database (HGMD): 2003 update. *Hum. Mutat.* **21**, 577–581 (2003).
40. Sherry, S. T., Ward, M. & Sirotkin, K. dbSNP-database for single nucleotide polymorphisms and other classes of minor genetic variation. *Genome Res.* **9**, 677–679 (1999).
41. Solomon, B. D., Nguyen, A.-D., Bear, K. A. & Wolfsberg, T. G. Clinical genomic database. *Proc. Natl. Acad. Sci. U. S. A.* **110**, 9851–9855 (2013).
42. Karczewski, K. J. *et al.* The mutational constraint spectrum quantified from variation in 141,456 humans. *Nature* **581**, 434–443 (2020).
43. Lek, M. *et al.* Analysis of protein-coding genetic variation in 60,706 humans. *Nature* **536**, 285–291 (2016).
44. 1000 Genomes Project Consortium *et al.* A global reference for human genetic variation. *Nature* **526**, 68–74 (2015).
45. Exome Variant Server. http://evs.gs.washington.edu/EVS/.
46. UK10K Consortium *et al.* The UK10K project identifies rare variants in health and disease. *Nature* **526**, 82–90 (2015).
47. Scott, E. M. *et al.* Characterization of Greater Middle Eastern genetic variation for enhanced disease gene discovery. *Nat. Genet.* **48**, 1071–1076 (2016).
48. Tadaka, S. *et al.* 3.5KJPNv2: an allele frequency panel of 3552 Japanese individuals including the X chromosome. *Hum Genome Var* **6**, 28 (2019).
49. GenomeAsia100K Consortium. The GenomeAsia 100K Project enables genetic discoveries across Asia. *Nature* **576**, 106–111 (2019).
50. Fattahi, Z. *et al.* Iranome: A catalog of genomic variations in the Iranian population. *Hum. Mutat.* **40**, 1968–1984 (2019).
51. MacDonald, J. R., Ziman, R., Yuen, R. K. C., Feuk, L. & Scherer, S. W. The Database of Genomic Variants: a curated collection of structural variation in the human genome. *Nucleic Acids Res.* **42**,

D986–92 (2014).
52. Lappalainen, I. *et al.* DbVar and DGVa: public archives for genomic structural variation. *Nucleic Acids Res.* **41**, D936–41 (2013).
53. Firth, H. V. *et al.* DECIPHER: Database of Chromosomal Imbalance and Phenotype in Humans Using Ensembl Resources. *Am. J. Hum. Genet.* **84**, 524–533 (2009).
54. Petrovski, S., Wang, Q., Heinzen, E. L., Allen, A. S. & Goldstein, D. B. Genic intolerance to functional variation and the interpretation of personal genomes. *PLoS Genet.* **9**, e1003709 (2013).
55. Gussow, A. B., Petrovski, S., Wang, Q., Allen, A. S. & Goldstein, D. B. The intolerance to functional genetic variation of protein domains predicts the localization of pathogenic mutations within genes. *Genome Biol.* **17**, 9 (2016).
56. Havrilla, J. M., Pedersen, B. S., Layer, R. M. & Quinlan, A. R. A map of constrained coding regions in the human genome. *Nat. Genet.* **51**, 88–95 (2019).
57. Hayeck, T. J. *et al.* Improved Pathogenic Variant Localization via a Hierarchical Model of Sub-regional Intolerance. *Am. J. Hum. Genet.* **104**, 299–309 (2019).
58. Traynelis, J. *et al.* Optimizing genomic medicine in epilepsy through a gene-customized approach to missense variant interpretation. *Genome Res.* **27**, 1715–1729 (2017).
59. Cassa, C. A. *et al.* Estimating the selective effects of heterozygous protein-truncating variants from human exome data. *Nat. Genet.* **49**, 806–810 (2017).
60. Fadista, J., Oskolkov, N., Hansson, O. & Groop, L. LoFtool: a gene intolerance score based on loss-of-function variants in 60 706 individuals. *Bioinformatics* **33**, 471–474 (2017).
61. Davydov, E. V. *et al.* Identifying a high fraction of the human genome to be under selective constraint using GERP++. *PLoS Comput. Biol.* **6**, e1001025 (2010).
62. Siepel, A. *et al.* Evolutionarily conserved elements in vertebrate, insect, worm, and yeast genomes. *Genome Res.* **15**, 1034–1050 (2005).
63. Adzhubei, I. A. *et al.* A method and server for predicting damaging missense mutations. *Nat. Methods* **7**, 248–249 (2010).
64. Ng, P. C. & Henikoff, S. SIFT: Predicting amino acid changes that affect protein function. *Nucleic Acids Res.* **31**, 3812–3814 (2003).
65. Schwarz, J. M., Rödelsperger, C., Schuelke, M. & Seelow, D. MutationTaster evaluates disease-causing potential of sequence alterations. *Nat. Methods* **7**, 575–576 (2010).
66. Smedley, D. *et al.* A Whole-Genome Analysis Framework for Effective Identification of Pathogenic Regulatory Variants in Mendelian Disease. *Am. J. Hum. Genet.* **99**, 595–606 (2016).
67. Rentzsch, P., Witten, D., Cooper, G. M., Shendure, J. & Kircher, M. CADD: predicting the deleteriousness of variants throughout the human genome. *Nucleic Acids Res.* **47**, D886–D894 (2019).
68. Ioannidis, N. M. *et al.* REVEL: An Ensemble Method for Predicting the Pathogenicity of Rare Missense Variants. *Am. J. Hum. Genet.* **99**, 877–885 (2016).
69. Quang, D., Chen, Y. & Xie, X. DANN: a deep learning approach for annotating the pathogenicity of genetic variants. *Bioinformatics* **31**, 761–763 (2015).
70. Jagadeesh, K. A. *et al.* M-CAP eliminates a majority of variants of uncertain significance in clinical exomes at high sensitivity. *Nat. Genet.* **48**, 1581–1586 (2016).
71. Quinodoz, M. *et al.* DOMINO: Using Machine Learning to Predict Genes Associated with Dominant Disorders. *Am. J. Hum. Genet.* **101**, 623–629 (2017).
72. Ionita-Laza, I., McCallum, K., Xu, B. & Buxbaum, J. D. A spectral approach integrating functional genomic annotations for coding and noncoding variants. *Nat. Genet.* **48**, 214–220 (2016).
73. Jaganathan, K. *et al.* Predicting Splicing from Primary Sequence with Deep Learning. *Cell* **176**, 535–548.e24 (2019).
74. Lord, J. *et al.* Pathogenicity and selective constraint on variation near splice sites. *Genome Res.* **29**, 159–170 (2019).
75. Jian, X., Boerwinkle, E. & Liu, X. In silico prediction of splice-altering single nucleotide variants in the human genome. *Nucleic Acids Res.* **42**, 13534–13544 (2014).
76. Desmet, F.-O. *et al.* Human Splicing Finder: an online bioinformatics tool to predict splicing signals. *Nucleic Acids Res.* **37**, e67 (2009).
77. Cheng, J. *et al.* MMSplice: modular modeling improves the predictions of genetic variant effects on

splicing. *Genome Biol.* **20**, 48 (2019).
78. Yeo, G. & Burge, C. B. Maximum entropy modeling of short sequence motifs with applications to RNA splicing signals. *J. Comput. Biol.* **11**, 377–394 (2004).
79. Gelfman, S. *et al.* Annotating pathogenic non-coding variants in genic regions. *Nat. Commun.* **8**, 236 (2017).
80. McLaren, W. *et al.* The Ensembl Variant Effect Predictor. *Genome Biol.* **17**, 122 (2016).
81. Wang, K., Li, M. & Hakonarson, H. ANNOVAR: functional annotation of genetic variants from high-throughput sequencing data. *Nucleic Acids Res.* **38**, e164 (2010).
82. Cingolani, P. *et al.* A program for annotating and predicting the effects of single nucleotide polymorphisms, SnpEff: SNPs in the genome of Drosophila melanogaster strain w1118; iso-2; iso-3. *Fly* **6**, 80–92 (2012).
83. Our Platform. https://www.genomoncology.com/our-platform.
84. Bozeman, MT: Golden Helix, Inc. *VarSeq™ [software]*.
85. Ng, S. B. *et al.* Targeted capture and massively parallel sequencing of 12 human exomes. *Nature* **461**, 272–276 (2009).
86. Bolger, A. M., Lohse, M. & Usadel, B. Trimmomatic: a flexible trimmer for Illumina sequence data. *Bioinformatics* **30**, 2114–2120 (2014).
87. Martin, M. Cutadapt removes adapter sequences from high-throughput sequencing reads. *EMBnet j.* **17**, 10 (2011).
88. Dobin, A. *et al.* STAR: ultrafast universal RNA-seq aligner. *Bioinformatics* **29**, 15–21 (2013).
89. Liao, Y., Smyth, G. K. & Shi, W. featureCounts: an efficient general purpose program for assigning sequence reads to genomic features. *Bioinformatics* **30**, 923–930 (2014).
90. Li, B. & Dewey, C. N. RSEM: accurate transcript quantification from RNA-Seq data with or without a reference genome. *BMC Bioinformatics* **12**, 323 (2011).
91. Love, M. I., Huber, W. & Anders, S. Moderated estimation of fold change and dispersion for RNA-seq data with DESeq2. *Genome Biol.* **15**, 550 (2014).
92. Robinson, M. D., McCarthy, D. J. & Smyth, G. K. edgeR: a Bioconductor package for differential expression analysis of digital gene expression data. *Bioinformatics* **26**, 139–140 (2010).
93. Brechtmann, F. *et al.* OUTRIDER: A Statistical Method for Detecting Aberrantly Expressed Genes in RNA Sequencing Data. *Am. J. Hum. Genet.* **103**, 907–917 (2018).
94. Li, Y. I. *et al.* Annotation-free quantification of RNA splicing using LeafCutter. *Nat. Genet.* **50**, 151–158 (2018).
95. Li, X. *et al.* The impact of rare variation on gene expression across tissues. *Nature* **550**, 239–243 (2017).
96. Birgmeier, J. *et al.* AMELIE speeds Mendelian diagnosis by matching patient phenotype and genotype to primary literature. *Sci. Transl. Med.* **12**, (2020).
97. Cipriani, V. *et al.* An Improved Phenotype-Driven Tool for Rare Mendelian Variant Prioritization: Benchmarking Exomiser on Real Patient Whole-Exome Data. *Genes* **11**, (2020).
98. James, R. A. *et al.* A visual and curatorial approach to clinical variant prioritization and disease gene discovery in genome-wide diagnostics. *Genome Med.* **8**, 13 (2016).
99. Gonzalez, M. *et al.* Innovative genomic collaboration using the GENESIS (GEM.app) platform. *Hum. Mutat.* **36**, 950–956 (2015).
100. Girdea, M. *et al.* PhenoTips: patient phenotyping software for clinical and research use. *Hum. Mutat.* **34**, 1057–1065 (2013).
101. Köhler, S. *et al.* Clinical diagnostics in human genetics with semantic similarity searches in ontologies. *Am. J. Hum. Genet.* **85**, 457–464 (2009).
102. Ekawade, A. *et al.* Genepanel.iobio - an easy to use web tool for generating disease- and phenotype-associated gene lists. *BMC Med. Genomics* **12**, 190 (2019).
103. Rubinstein, W. S. *et al.* The NIH genetic testing registry: a new, centralized database of genetic tests to enable access to comprehensive information and improve transparency. *Nucleic Acids Res.* **41**, D925–35 (2013).
104. Yang, H., Robinson, P. N. & Wang, K. Phenolyzer: phenotype-based prioritization of candidate genes for human diseases. *Nat. Methods* **12**, 841–843 (2015).
105. Shefchek, K. A. *et al.* The Monarch Initiative in 2019: an integrative data and analytic platform

connecting phenotypes to genotypes across species. *Nucleic Acids Res.* **48**, D704–D715 (2020).
106. Bult, C. J. *et al.* Mouse Genome Database (MGD) 2019. *Nucleic Acids Res.* **47**, D801–D806 (2019).
107. Köhler, S. *et al.* Expansion of the Human Phenotype Ontology (HPO) knowledge base and resources. *Nucleic Acids Res.* **47**, D1018–D1027 (2019).
108. Guzman, J. *et al.* The Undiagnosed Diseases Program Integrated Collaboration System (UDPICS): One Program's Experience Developing Custom Software to Support Research for Complex-Disease Families. *Children* **2**, 342–357 (2015).
109. Robinson, P. N. *et al.* Improved exome prioritization of disease genes through cross-species phenotype comparison. *Genome Res.* **24**, 340–348 (2014).
110. Harris, P. A. *et al.* Research electronic data capture (REDCap)--a metadata-driven methodology and workflow process for providing translational research informatics support. *J. Biomed. Inform.* **42**, 377–381 (2009).
111. Paila, U., Chapman, B. A., Kirchner, R. & Quinlan, A. R. GEMINI: integrative exploration of genetic variation and genome annotations. *PLoS Comput. Biol.* **9**, e1003153 (2013).
112. Haghighi, A. *et al.* An integrated clinical program and crowdsourcing strategy for genomic sequencing and Mendelian disease gene discovery. *NPJ Genom Med* **3**, 21 (2018).
113. Griffith, M. *et al.* Genome Modeling System: A Knowledge Management Platform for Genomics. *PLoS Comput. Biol.* **11**, e1004274 (2015).
